# Influence of Glycine and Arginine on Cylindrospermopsin Production and *aoa* Gene Expression in *Aphanizomenon ovalisporum*

**DOI:** 10.3390/toxins9110355

**Published:** 2017-11-01

**Authors:** Ángel Barón-Sola, Francisca Fernández del Campo, Soledad Sanz-Alférez

**Affiliations:** Departament of Biology, Universidad Autónoma de Madrid, Campus de Cantoblanco, 28049 Madrid, Spain; angel.baron@uam.es (Á.B.-S.); francisca.delcampo@uam.es (F.F.d.C.)

**Keywords:** *Aphanizomenon ovalisporum*, *aoa*/*cyr*/*ntcA* genes expression, guanidinoacetate, cylindrospermopsin, arginine, glycine

## Abstract

Arginine (Arg) and glycine (Gly) seem to be the only substrates accepted by the amidinotransferase that catalyze the first step of the synthesis pathway of the cyanotoxin cylindrospermopsin (CYN), leading to guanidinoacetate (GAA). Here, the effect of these amino acids on the production of CYN in cultures of the cylindrospermopsin-producing strain, *Aphanizomenon ovalisporum* UAM-MAO, has been studied. Arg clearly increased CYN content, the increment appearing triphasic along the culture. On the contrary, Gly caused a decrease of CYN, observable from the first day on. Interestingly, the transcript of the gene *ntcA*, key in nitrogen metabolism control, was also enhanced in the presence of Arg and/or Gly, the trend of the transcript oscillations being like that of *aoa*/*cyr*. The inhibitory effect of Gly in CYN production seems not to result from diminishing the activity of genes considered involved in CYN synthesis, since Gly, as Arg, enhance the transcription of genes *aoaA-C* and *cyrJ.* On the other hand, culture growth is affected by Arg and Gly in a similar way to CYN production, with Arg stimulating and Gly impairing it. Taken together, our data show that the influence of both Arg and Gly on CYN changes seems not to be due to a specific effect on the first step of CYN synthesis; it rather appears to be the result of changes in the physiological cell status.

## 1. Introduction

There is a correlation between eutrophication, climate change, and toxic harmful algal blooms (HABs), in which cyanobacteria are significant components. A high concentration of nitrogen (N) and phosphorous (P) contributes to the massive proliferation of toxic cyanobacteria in water reservoirs for consumption or recreational purposes [1]. Among the cyanotoxins, the potent alkaloid and protein synthesis inhibitor cylindrospermopsin (CYN) is of increasing concern, due to the growing number of world-wide detections reported in recent years [2]. Inorganic nutrient availability seems to play a key role in CYN production, since nitrogen, phosphate, and sulphate starvations cause significant changes in toxin production [3,4,5,6].

Despite the large molecular information related to CYN production, data about the regulation of its synthesis is still scarce. To date, all gene clusters associated with CYN synthesis (*aoa*/*cyr*) are highly similar along all the gene regions. The CYN synthesis pathway model includes an amidinotransferase (AMDT), codified by the *aoaA*/*cyrA* gene, that would catalyze the first step to produce guanidinoacetic acid (GAA). In following steps, the putative enzymes involved would be: a mixed enzyme complex constituted by a non-ribosomal peptide synthetase (NRPS)-polyketide synthase (PKS), codified by *aoaB*/*cyrB*; a PKS codified by *aoaC*/*cyrC*; other PKSs; and tailoring proteins. AMDTs of the CYN-producing strains *Cylindrospermopsis raciborskii* AWT205 (CyrA) [7] and *Aphanizomenon ovalisporum* UAM-MAO (AoaA) [8] were cloned, overexpressed, and biochemically characterized. Unlike the AMDTs described before, CyrA and AoaA show narrow substrate specificity, seeming to work well just with arginine (Arg) and glycine (Gly), as an amidino group donor and acceptor, respectively. In addition, a complex mixed ping-pong kinetic mechanism is involved in the two cyanobacteria AMDTs.

*A. ovalisporum* is a bloom-forming cyanobacterium, being CYN producers with all its reported strains, except one [9]. Recently, it was described as a potential invasive species [10,11]. Interestingly, it has been reported that different organic nitrogen compounds can be utilized by *A. ovalisporum* ILC146 strain, being the major N source in *A. ovalisporum* bloom episodes in Lake Kinneret, Israel [12]. The role of organic nitrogen compounds, amino acids among them—on cyanobacterial growth and on primary and secondary metabolism—has been deeply studied in various cyanobacteria species [13,14,15,16].

Regarding Arg and Gly, the specific substrates of the cyanobacteria AMDTs responsible for the first step of CYN synthesis, we thought it interesting to explore if these amino acids influenced CYN production. For that, the production of CYN and GAA in cultures of *A. ovalisporum* UAM-MAO grown in the absence and in the presence of Arg and/or Gly was compared. As well, the effect of these amino acids on the expression of four genes involved in CYN formation, *aoaA-C* and *cyrJ*, were compared. Furthermore, the influence of Arg and Gly was also evaluated in the expression of the gene, *ntcA*, that codifies for a key regulator of nitrogen metabolism; this considers the high nitrogen content of CYN, and the previous suggestion [8] of a possible role of that gene in CYN production.

## 2. Results

### 2.1. Effect of Arginine and Glycine on Growth 

Growth was followed by the absorbance at 750 nm and Chl*a* concentration, but under the conditions used, the expected growth curve was only obtained considering the absorbance values. Therefore, the absorbance was the elected growth parameter.

**Figure 1:**
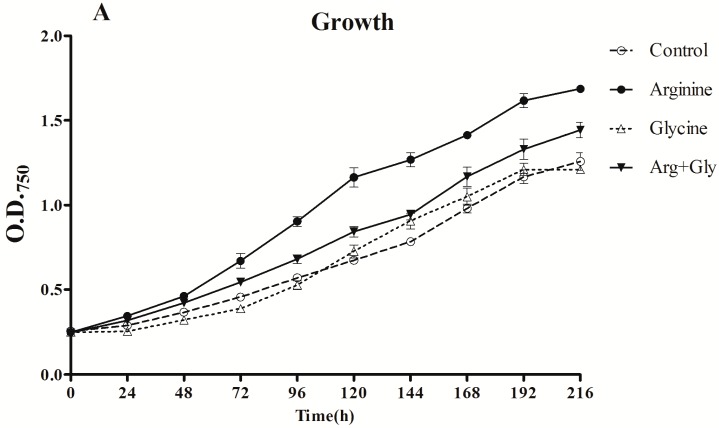

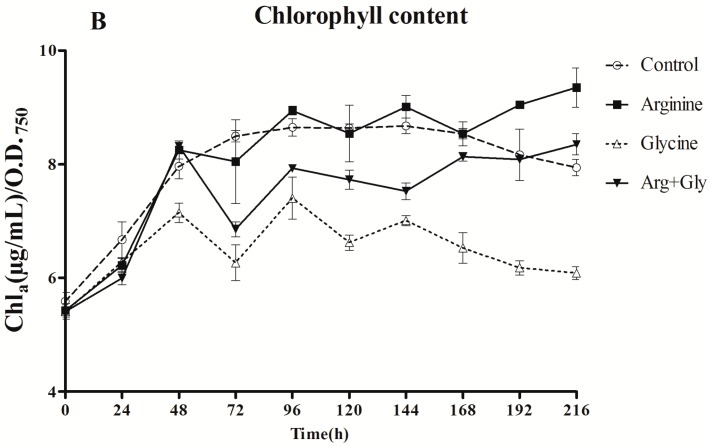
Effect of arginine and glycine on (**A**) growth and (**B**) chlorophyll concentration of *A. ovalisporum* UAM-MAO. Values are the average of three replicates; error bars indicate ±SD from the mean (*n* = 3).

The cultures with added Arg, either alone or with Gly, grew significantly (*p* < 0.01) better than the control or Gly cultures, with the strongest stimulatory effect being when Arg was added alone (Figure 1). Remarkably, the growth in the culture supplemented with Gly was the lowest in a significant way (*p* < 0.01) during the first half of the assay (24–96 h).

Chl*a* content was affected in a different way by the presence of Arg and Gly. The effect of Arg supplementation was not significant (*p* < 0.01) until the two last samplings, when a stimulatory effect was observed. However, Gly clearly caused a considerable reduction in Chl*a* concentration throughout the assay (*p* < 0.01). The addition of both amino acids also significantly decreased (*p* < 0.01) Chl*a* concentration with respect to the control, but the decrease was less pronounced than in Gly cultures.

### 2.2. CYN Accumulation

**Figure 2:**
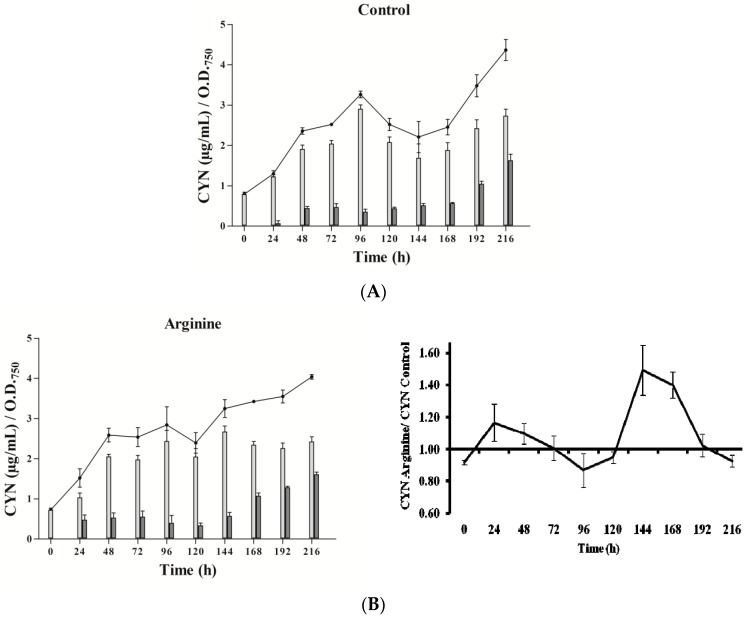

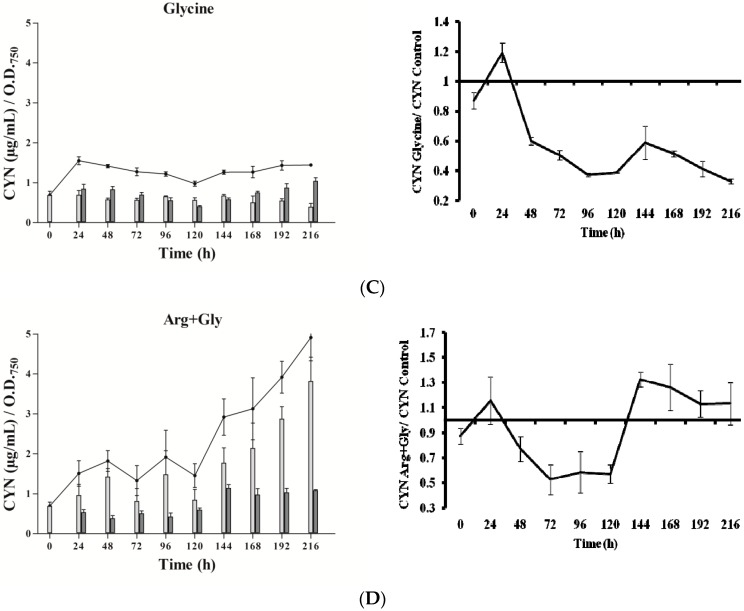
Effect of arginine and glycine on cylindrospermopsin content in *A. ovalisporum* UAM-MAO. (**A**) Control culture; (**B**) plus Arg; (**C**) plus Gly; (**D**) plus Arg and Gly. Total cyanotoxin cylindrospermopsin (CYN) is plotted in lines; intracellular and extracellular CYN is in light and dark grey bars, respectively. Values are the average of three replicates; error bars indicate ± SD from the mean (*n* = 3).

Three phases in CYN accumulation were appreciated, along the experimental time (216 h), in the three types of cultures utilized (Figure 2). Initially, an increase of the toxin content was detected, followed by an intermediate phase with a CYN reduction and a final stage, with a new toxin rise. The duration and intensity of these phases were quite different between cultures.

In the presence of Arg (Figure 2B), CYN content did not differ remarkably from the control (Figure 2A). Gly addition caused a significant increase of CYN just at 24 h, followed by an almost steady level (Figure 2C), with values always lower than the control. Arg plus Gly supplementation caused a slight increase in toxin content (Figure 2D), followed by several fluctuations until 120 h, when CYN rose constantly. In general, intracellular CYN was the main fraction, representing 65–90%; an exception was the glycine-supplemented culture, in which the intracellular fraction was only 30–50%. Remarkably, in this Gly culture, the percentage of extracellular CYN rose in the last points assayed (seventh to ninth day).

### 2.3. Gene Expression Analysis 

**Figure 3:**
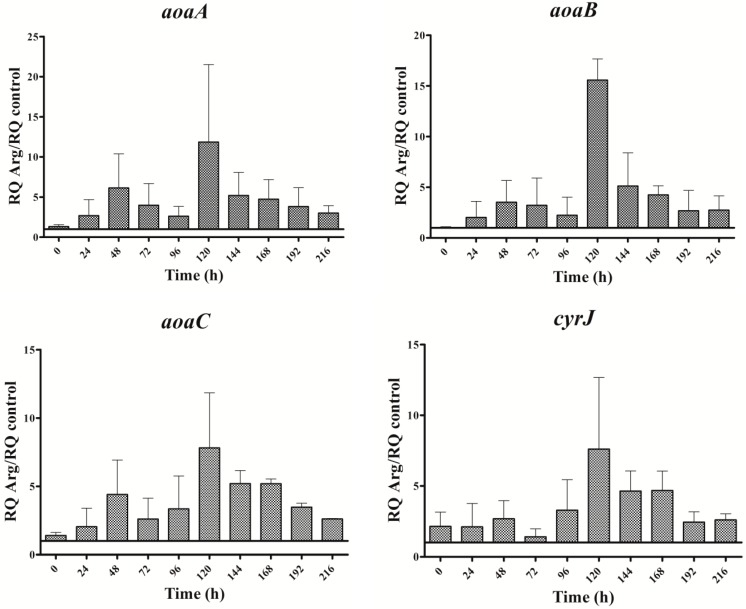
Relative expression of *aoaA*, *aoaB*, *aoaC*, and *cyrJ* genes in *A. ovalisporum* UAM-MAO grown in BG11 medium supplemented with arginine. Data are presented as the ratio between expression levels in Arg supplemented (BG11 + Arg) and control culture (BG11). The expression levels are relative to 16S rRNA gene. Error bars indicate standard deviations of three replicates.

The expression of all genes analyzed throughout the assay time was significantly higher in the Arg culture than in the control (Figure 3). The oscillation pattern of the *aoa* and *cyr* transcripts was similar. The transcription increased initially (from 0 to 48 h), then declined (until 72 h in *aoaC* and *cyrJ* or 96 h in *aoaA-B*); later, it increased again, reaching the highest value at 120 h; and finally, a gradual decrease was observed. This oscillatory pattern was different from that of CYN content (cf. Figure 2B and Figure 3). In effect, CYN content in the Arg-supplemented medium increased (*p* < 0.01) slightly with respect to the control at 24, and largely by about 150 h (Figure 2B). A small gene expression increase coincided with the first rise of CYN; however, the second increase of gene expression took place when CYN was leveling off. Moreover, the biggest CYN peak appeared 24 h later than that of gene expression.

**Figure 4:**
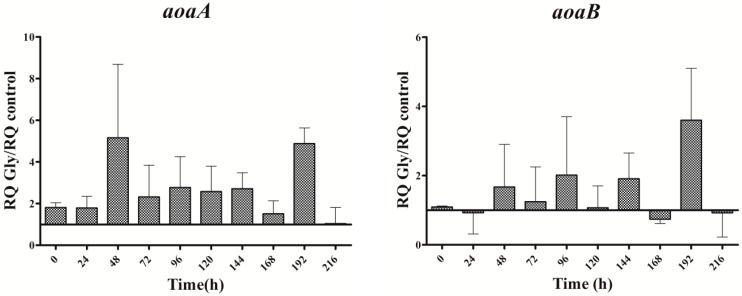

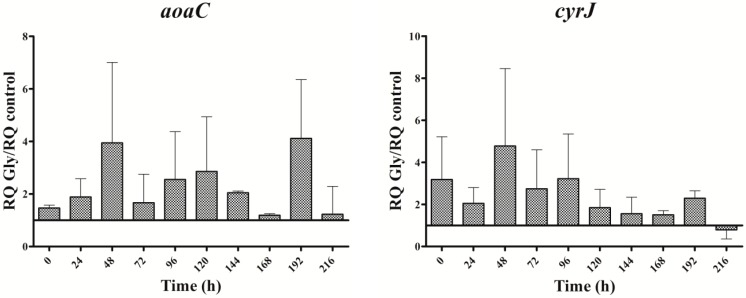
Relative expression of *aoaA*, *aoaB*, *aoaC*, and *cyrJ* genes in *A. ovalisporum* UAM-MAO strain grown in BG11 medium supplemented with glycine. Data are presented as the ratio between expression levels in Gly supplemented (BG11 + Gly) and in the control culture (BG11). The expression levels are relative to 16S rRNA gene. Error bars indicate standard deviations of three independent experiments.

In cultures supplemented with Gly alone, the gene transcripts increased with respect to the control, except for the *aoaB* gene, which showed some repression data at several times along the assay (Figure 4). Unlike Arg cultures, the increment pattern differed among the transcripts. Thus, the maximum of *aoaA*, *aoaC*, and *cyr* transcripts was at 48 h, while that of *aoaB* was at 192 h. The *aoaA* and *aoaC* expression also showed a high increment at 192 h. Curiously, *aoaB* expression maintained an almost cycling pattern. The effect of Gly on *aoa* genes transcription contrasts, therefore, with that on CYN production (Figure 2C), since the toxin content was significantly lower (*p* < 0.01) than that of the control from 24 h to the end (216 h). Only at 24 h was the content considerably higher in the Gly-supplemented culture than in the control.

**Figure 5:**
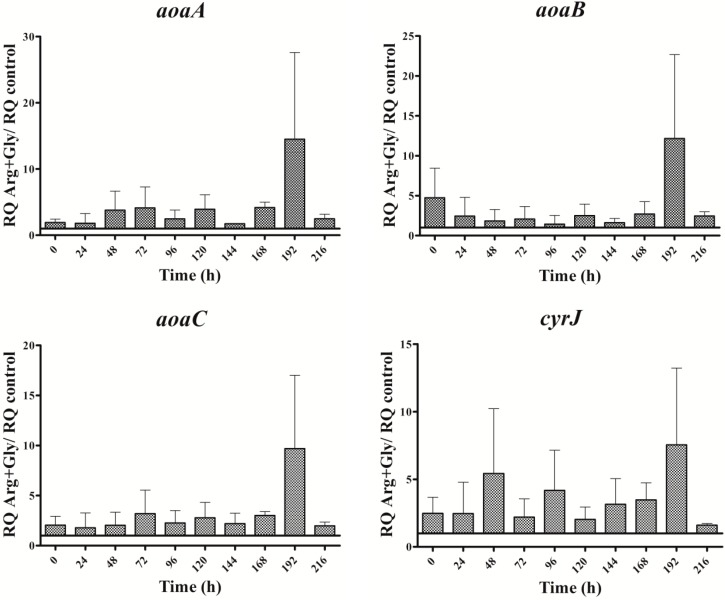
Relative gene expression of *aoaA*, *aoaB*, *aoaC*, and *cyrJ* genes in *A. ovalisporum* UAM-MAO strain grown in BG11 medium supplemented with arginine and glycine. Data are presented as the ratio between the expression levels in the Arg plus Gly-supplemented culture and the control culture (BG11). The expression levels are relative to 16S rRNA gene. Error bars indicate standard deviations of three replicates.

In the presence of Arg plus Gly, *aoa*, and *cyr* transcript levels were also bigger than in the control (Figure 5). The fluctuations of all the transcripts were similar throughout the time, with a maximum at 192 h. From 0 to 192 h, a continuous up and down was observed; however, there were differences between the genes, both with respect to time duration and intensity. Interestingly, the CYN content variations exhibited a distinct trend (Figure 2D). During the first half of the experiment a similar tendency to that of Gly treatment was observed, while during the second part, the pattern was closer to that of Arg-supplemented cultures.

### 2.4. GAA Accumulation

**Figure 6:**
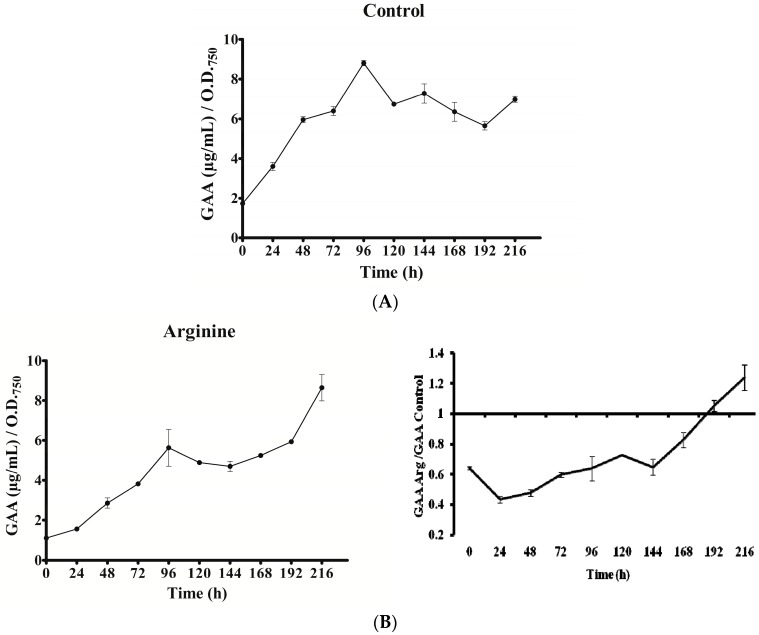

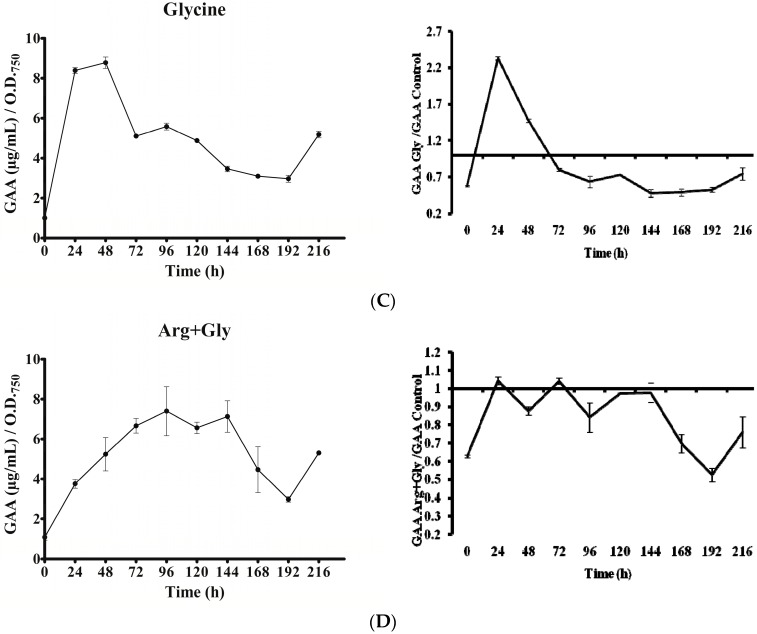
Effect of arginine and glycine on intracellular guanidinoacetate of *A. ovalisporum* UAM-MAO. (**A**) Control culture; (**B**) Arg-supplemented culture; (**C**) Gly-supplemented culture; (**D**) Arg plus Gly-supplemented culture. Values are the average of three replicates; error bars indicate ±SD from the mean (*n* = 3).

In all cultures, intracellular GAA varied with time (Figure 6). Except in the Gly culture, a gradual increase of GAA was observed from 0 to 96 h. Nevertheless, the rise slope as well as the maximum value attained differed. In the Gly culture, GAA rose abruptly within the first 24 h. In all the cases, after the initial increase, a long decrease was observed until the penultimate day of the experiment (192 h). The GAA value remained in the Arg and Arg plus Gly cultures below the control until that day, when GAA content rose again conspicuously, above the control value. Interestingly, in all instances the concentration of GAA (amount per biomass unit) was 1.5 to 9 times higher than that of CYN. No correlation was observed between GAA fluctuations and transcription of any of the *aoa*/*cyr* genes (Figure 3, Figure 4, Figure 5 and Figure 6).

### 2.5. Gene Expression Analysis of *ntcA*

**Figure 7:**
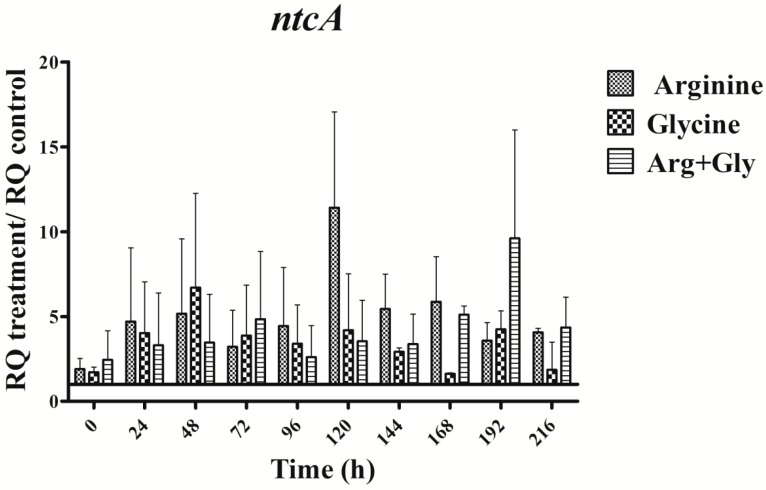
Relative expression of *ntcA* gene in *A. ovalisporum* UAM-MAO grown in BG11 medium supplemented with arginine and/or glycine. Data are presented as the ratio between the expression levels in the amino acid-supplemented cultures and the control culture (BG11). The expression levels are relative to 16S rRNA gene. Error bars indicate standard deviations of three replicates.

The expression of the nitrogen master control gene *ntcA* was enhanced throughout the time in all treatments assayed (Figure 7). In every amino acid treatment gene expression changes were observed, but the changes were different in duration and magnitude, and depended on the gene studied. Interestingly, the trend of the *ntcA* transcript changes pattern was like that of the *aoa*/*cyr* genes (Figure 3, Figure 4 and Figure 5 and Figure 7).

## 3. Discussion

We have evaluated in *A. ovalisporum* UAM-MAO the influence of Arg and Gly in CYN production, considering that these amino acids could favor the synthesis of the toxin in two manners: first, by providing the unique substrates apparently recognized by the AMDT responsible for the GAA formation in the first step of CYN synthesis [7]; and second, by enhancing available nitrogen, which would contribute to satisfying the high N needs for CYN synthesis. The experiments have been performed in batch cultures of the CYN+ *A. ovalisporum* strain UAM-MAO, grown in a medium with NO3^−^ as initial N source (BG11), supplemented or not with Arg, Gly, or a mixture of both amino acids.

CYN content was affected in a different way by the presence of the amino acids: Arg clearly enhanced the production, while Gly decreased it (Figure 2) after the first 24 h. Both increase and decrease were concentration-dependent (data not shown). The inhibition by Gly was alleviated by the presence of Arg. The enhancement by Arg exhibited a curious triphasic pattern.

It appears that the influence by Arg and Gly in CYN production could not be attributed to effects on the expression of the genes related to its synthesis, as the transcription of the *aoaA-C* and *cyrJ* genes are similarly increased by either of the two amino acids (Figure 3, Figure 4 and Figure 5).

Regarding *aoaA*, the gene codifying for the AMDT that catalyzes the formation of GAA from Arg and Gly, special emphasis has been put on comparing the effect of the two amino acids on both *aoaA* expression and GAA production. No correlation was found between the *aoaA* transcript levels and GAA cell content in the different cultures utilized (cf. Figure 3, Figure 4, Figure 5 and Figure 6). Arg and Gly when added alone or together significantly increased *aoaA* gene transcription throughout the experimental period; but GAA content was significantly lower in the Arg in all treatments with respect to the control, except in part of the last growth period. GAA increased during the first 48 h upon Gly addition; later it leveled off, in parallel to CYN. Arg treatment caused different GAA increments along the culture, in a triphasic way like CYN (cf. Figure 2 and Figure 6). Despite the basis found to think that the control by Arg and Gly of CYN and GAA production is not at a transcriptional level, other genes in the *aoa* cluster from *A. ovalisporum* UAM-MAO should be analyzed.

It has been previously observed that different nitrogen sources affect CYN content [3,5,17] and *aoa*/*cyr* expression levels [5,17], but no experiment to analyze the effect of amino acids was performed. It would be advisable to perform experiments with other CYN+ strains from different species, as well as with other amino acids, to assess how the presence of a specific amino acid could condition CYN production.

**Figure 8:**
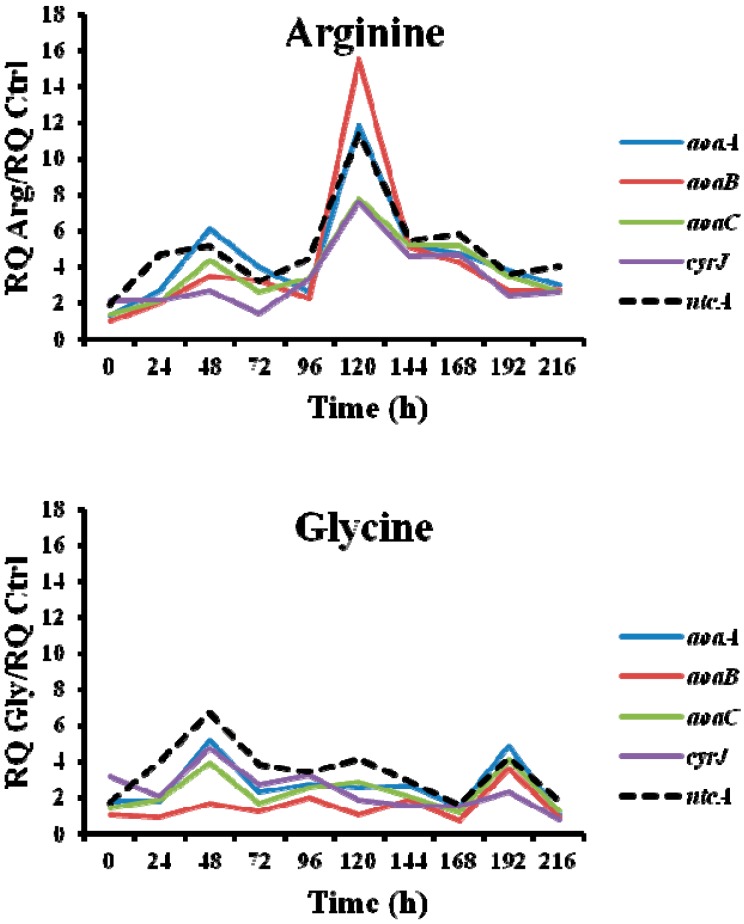

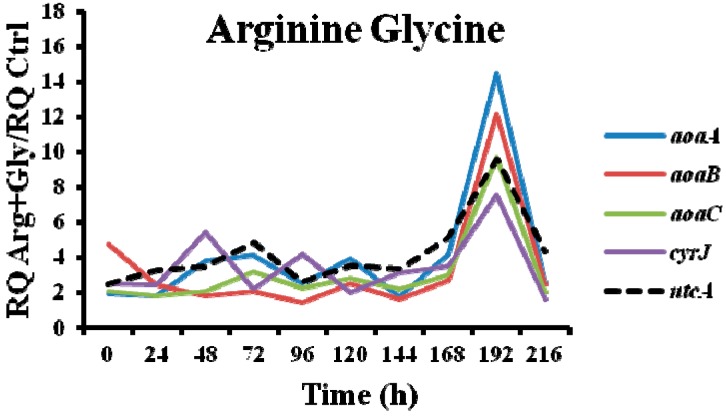
Relative expression of *aoaA*, *aoaB*, *aoaC*, *cyrJ*, and *ntcA* genes in *A. ovalisporum* UAM-MAO strain grown in BG11 medium supplemented with arginine and/or glycine. Data are presented as the ratio between the expression levels in the amino acid-supplemented cultures and the control culture (BG11). The expression levels are relative to 16S rRNA gene.

Interestingly, the transcription of the gene *ntcA* was significantly enhanced with respect to the control in the presence of all the amino acids assayed (Figure 7), the trend of the transcription kinetics being similar that of *aoa*/*cyr* (Figure 8). That fact suggests, among other ideas, that *ntcA* and the genes responsible for CYN synthesis are controlled by a common regulator, and/or that *ntcA* controls *aoaA* and *cyrJ* expression. Previous analyses of *ntcA* transcription under different nitrogen sources showed that ammonium caused the repression of this gene, while other nitrogen compounds increased it [18,19]. Besides, the assimilation of different organic nitrogen compounds is controlled by *ntcA* [20]. Further experiments are needed to clarify how Arg and Gly modulate in a similar way the activity of *ntcA*, *aoaA-C*, and *cyrJ* genes.

The influence of both Arg and Gly on CYN in *A. ovalisporum* UAM-MAO appears to be the result of changes in the physiological cell status, since its growth was remarkably favored in the presence of Arg, and reduced with Gly. Besides, with the mixture of both amino acids an intermediate growth effect was observed, as it occurred with CYN production (Figure 1 and Figure 2).

The growth stimulation by Arg was not a surprise, since it was already established that amino acids and other organic nitrogen sources can be used by cyanobacteria as a source of N and C [13,21,22]. With respect to utilization of Arg by *A. ovalisporum*, it was also reported that dissolved organic nitrogen was the main N source utilized by a strain of this species in a bloom episode in Lake Kinneret, and that other organic nitrogen compounds, including some amino acids, could serve for its growth [12]. Amino acid transporters with high and low affinity for Arg have been described in detail in the filamentous heterocyst-forming cyanobacteria *Anabaena* sp. PCC 7120 [22]. Besides, we have observed a significant reduction of heterocyst density in *A. ovalisporum* UAM-MAO (data not shown) when Arg was used as the only N source. It has been also demonstrated that simultaneous assimilation of amino acids (including arginine), ammonium, and nitrate can take place [23]. Moreover, large intracellular accumulation of Arg was described in *Synechocystis* sp. 6803 [24], suggesting an important physiological role of this amino acid in cyanobacteria, apart from its role as source for nitrogen reserve-forming cyanophycine from aspartate.

Growth stimulation and reduction of *A. ovalisporum* UAM-MAO by Arg and Gly, respectively, seem to be dose dependent (data not shown). When planning this work, toxicity by Gly was unexpected due to its utilization as a substrate for CYN production; however, amino acid toxicity has been reported in several cyanobacteria [21,22,23,24,25,26]. Specifically, Gly accumulation has been described as toxic for *Synechocystis* sp. strain PCC6803 [27], and *Anabaena cylindrica* PCC7120 [28]; also Gly is not useful for *Nostoc muscorum* growth [29]. This amino acid acts as a potent chelating agent for bivalent cations; it was described that addition of MgCl2 to the medium reduced glycine toxicity, suggesting that Gly reduces the number of bivalent cations, particularly Mg^2+^ [26,27]. That could be the reason of the significant lowering of Chl*a* content in the Gly and Arg + Gly treatments (Figure 1). On the other hand, the halotolerant cyanobacteria *Aphanothece halophytica* uses Gly as a precursor to the glycine betaine synthesis, being able to grow even at a highly toxic level of Gly at 60 mM [30], in which most microorganism show growth inhibition [31]. The toxicity of Gly has been related to membrane permeability in *Escherichia coli* [32], and with the activity of glycine descarboxylase in *Arabidopsis thaliana* [33]; however, the mechanism of Gly toxicity in cyanobacteria remains unknown.

## 4. Conclusions

Taken together, the data herein reveal that CYN and GAA production are clearly affected by their presence in the medium of Arg and Gly, and that the effects produced could be the result of the amino acid metabolism, and not necessarily of a direct action on AMDT activity.

The presence of arginine and glycine, both being an N source and substrates of the cyanobacterial AMDTs CyrA and AoaA, affect CYN and GAA production by *A. ovalisporum* UAM-MAO; however, each amino acid does it in a different manner. Thus, Arg promotes the production of the two compounds, but Gly diminishes it. Curiously, the two amino acids stimulate the transcription of *aoaA-C*, *cyr J*, and *ntcA* genes. Growth and chlorophyll *a* content is affected by Arg and Gly in a similar way to CYN content. Taken together, our data suggest that CYN and GAA production can be regulated postranscriptionally, and that the cell physiological status plays a key role in the production of the two compounds. Moreover, the amino acid effects observed in our experiments could also occur in nature, since dissolved organic nitrogen has been described as an important source of carbon and nitrogen for cyanobacterial growth in aquatic ecosystems [12,34]. Furthermore, all cyanobacterial strains studied to date present transporters for organic nitrogen molecules including amino acids.

## 5. Materials and Methods

### 5.1. Culture Conditions

*A. ovalisporum* UAM-MAO strain [35] was used. Three independent experiments were performed with batch BG11 [36] (pH 8; 20 mM HEPES) cultures, at 28 °C, with continuous white light of 60 μmol photons m-2 s-1, and constant aeration. Four different culture conditions were assayed: a control, with nitrate as nitrogen source (BG11), and three cultures supplemented with 1 mM Arg, Gly, or both. Samples were withdrawn every 24 h for nine days, and all of them analyzed in triplicate. Cell observations were done with an Olympus BH-2 microscope at 400× magnification equipped with a Leica DC300F digital system.

### 5.2. Growth Parameters

Growth was followed by measuring the O.D. at 750 nm. Chlorophyll *a* (Chl*a*) was also determined colorimetrically, essentially after Marker et al. method (1980) [37].

### 5.3. Gene Expression Analysis

The transcription analysis of different genes (*aoaA-C*, *cyrJ*, and *ntcA*) was carried out as described in Barón et al. (2013) [8]. Gene expression data from the real-time qPCR amplification were evaluated using the Ct value, and the 16S RNA gene was used as control gene to normalize the expression levels of target genes. Relative transcription was calculated using the 2-DDCt method, according to the handbook of Fast Real-Time Cycler-Applied Biosystems.

### 5.4. CYN and GAA Determination 

CYN was determined as in Barón et al. 2013 [8] and quantified in samples taken every 24 h for nine days. Extracellular GAA in the cultures could not be directly determined, due to the interference of the culture medium salts in the analysis; however, dissolved GAA could be quantified after discarding the culture medium by centrifugation and re-suspending the cells in water; it was quantified as in Barón et al. 2015 [38].

### 5.5. Data Analysis 

All data analyses were performed using GraphPad Prism^®^ 5 (GraphPad, La Jolla, CA, USA) and statistical analyses ANOVA (Bonferroni test) were performed using SPSS software^®^ (version 19.0.0, Armonk, NY, USA, 2011).
